# Impacts of anti‐inflammatory phosphodiesterase inhibitors on a murine model of chronic pulmonary inflammation

**DOI:** 10.1002/prp2.840

**Published:** 2021-07-29

**Authors:** Xiao‐Fang Zheng, Dan‐Dan Chen, Xiao‐Ling Zhu, Jehane Michael Le Grange, Lu‐Qian Zhou, Jin‐Nong Zhang

**Affiliations:** ^1^ Department of Emergency Medicine Union Hospital Tongji Medical College Huazhong University of Science and Technology Wuhan China; ^2^ Guangzhou Institute of Respiratory Health The First Affiliated Hospital of Guangzhou Medical University Guangzhou China

**Keywords:** cigarette smoke, histone deacetylase‐2, lung inflammation, phosphodiesterase inhibitors

## Abstract

Chronic obstructive pulmonary disease (COPD) often tends to respond poorly to glucocorticoid (GC) therapy. Reduced Histone deacetylase‐2 (HDAC‐2) activity is an important mechanism behind this GC insensitivity. In this study, we investigated the effects of three phosphodiesterase inhibitors (PDEIs), with an anti‐inflammatory propensity, on cigarette smoke (CS)‐induced pulmonary inflammation and HDAC‐2 activity. Male C57BL/6 mice were exposed to cigarette smoke (CS) over the course of 30 weeks. Administration of the PDEIs commenced from the 29th week and followed a schedule of once daily treatments, 5 days a week, for 2 weeks. Roflumilast (ROF) was administered intragastrically (5 mg·kg^−1^), while pentoxifylline (PTX) (10 mg·kg^−1^) and theophylline (THEO) (10 mg·kg^−1^) were administered intraperitoneally, either alone or in combination with a GC (triamcinolone acetonide or TRI, 5 mg·kg^−1^, i.m., single injection). Lung morphometry, as well as the activity of HDAC‐2, pro‐inflammatory cytokines and reactive oxygen species (ROS) were assessed at the end of the 30‐week course. CS exposure was associated with a reduction in HDAC‐2 activity and the up‐regulation of ROS expression. PTX, ROF, and THEO administration led to the partial restoration of HDAC‐2 activity, which was favorably associated with the reduction of ROS expression. However, combining TRI to any of these PDEIs did not synergistically augment HDAC‐2 activity. Inactivation of HDAC‐2 due to long‐term CS exposure is closely related to exaggerated oxidative stress, and this reduced HDAC‐2 activity could partially be restored through the use of PDEIs. This finding provides a potential novel approach for further clinical research.

## Graphical abstract

Long‐term cigarette smoke exposure is associated with a reduction in histone deacetylase‐2 (HDAC‐2) activity and the up‐regulation of IL‐8, TNF‐a and reactive oxygen species (ROS) expressions. Pentoxifylline (PTX), roflumilast (ROF) or theophylline (THEO) administration partially restores the HDAC‐2 activity, which is favorably associated with the reduction of ROS expression.

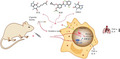

**Abbreviations**
BALF: bronchoalveolar lavage fluid
COPD: chronic obstructive pulmonary disease
CS: cigarette smoke
HDAC‐2: histone deacetylase‐2
Lm: mean linear intercept
PTX: pentoxifylline
ROF: roflumilast
ROS: reactive oxygen species
THEO: theophylline
TRI: Triamcinolone acetonide

## 1 INTRODUCTION

Chronic obstructive pulmonary disease (COPD) affects approximately 13% of people over the age of 40^1^ and was the fifth leading cause of death in China in 2016.^2^ The 2021 Global Initiative for Chronic Obstructive Lung Disease report,^3^ suggests that current pharmacological therapy for COPD is far from optimal and there is no conclusive evidence that any existing medication modifies disease progression in COPD patients, leading to a long‐term decline in lung function. Unlike bronchial asthma, in which patients could possibly control using glucocorticoid steroids (GCs), the response to GCs treatment in patients with COPD is not as effective as in asthma. This insensitivity to GCs in COPD patients is thought to be due to a reduction in histone deacetylase‐2 (HDAC2) activity, which correlates with COPD clinical severity.^4^, ^5^, ^6^ Excessive inflammation, together with enhanced oxidative stress, is an important mechanism for this decrease in HDAC‐2 activity in patients with COPD.^5^, ^7^ Restoration of GC sensitivity is postulated as a novel approach in COPD management.^8^, ^9^

Theophylline (THEO), pentoxifylline (PTX), and roflumilast (ROF) are three commonly prescribed phosphodiesterase (PDE) inhibitors as anti‐inflammatory treatments. THEO is prescribed as a bronchodilator, while PTX is indicated in occlusive peripheral artery disease (intermittent claudication).^10^ Of particular interest is that PTX has specific inhibitory effects on tumor necrotic factor (TNF)‐α, as the latter plays an important role in the pathogenesis of pulmonary inflammation induced by cigarette smoke (CS). Having been used for many years, PTX is also well known by clinicians for its safety and well toleration, even in long‐term clinical use. ROF is a PDE4 inhibitor approved by the FDA for the treatment of adults with severe COPD.^11^, ^12^

Certain laboratory studies have claimed that GC sensitivity could be re‐established by using THEO to up‐regulate HDAC‐2 expression,^13^, ^14^ however adding THEO to inhaled GCs did not result in improved clinical outcomes.^15^ We consider whether other anti‐inflammatory PDE inhibitors (PDEIs), such as PTX and ROF, had a similar or even greater effect on restoring GC‐sensitivity by restoring HDAC‐2 activity, thereby providing a novel avenue for clinical investigation.

## 2 MATERIALS AND METHODS

### 2.1 Study design

To compare the effects of THEO, PTX, and ROF when used alone and in combination with a GC, the mice were randomly divided into nine groups (*n* = 10 for each group): (1) Sham CS exposure; (2) CS exposure; (3) CS exposure with ROF administration; (4) CS exposure with PTX administration; (5) CS exposure with THEO administration; (6) CS exposure with triamcinolone (TRI) administration; (7) CS exposure with the co‐administration of ROF and TRI; (8) CS exposure with the co‐administration of PTX and TRI; (9) CS exposure with the co‐administration of THEO and TRI. The course set for CS exposure was 30 weeks, and the medication intervention commenced from the 29th week of the course and continued for 2 weeks.

### 2.2 Animals

Six‐ to seven‐week‐old male wild‐type C57BL/6 mice (18–22 g body weight) were purchased from Vital River Laboratory and Animals Co., Ltd.. The animals were housed in a specific pathogen‐free environment, at a temperature of 22–26℃ with a humidity of 60%–70%, where a 12‐h day/night cycle was maintained. The mice had free access to standard laboratory food and fresh water. The laboratory animal management rules of Tongji Medical College of Huazhong University of Science and Technology were strictly adhered to.

### 2.3 Cigarette smoke exposure

The animals in different groups were caged separately in a series of transparent chambers (each sized 28 × 21 × 17 cm) that were arranged in parallel and connected to a central CS supply, where smoke from the ignited cigarettes (Hongjinglong™) was automatically pumped evenly into each chamber. Each cigarette contained 10mg of tar, 0.8mg of nicotine, and 12 mg of carbon monoxide. During each 60‐min episode of smoke exposure, eight cigarettes without filters were combusted in turn, generating an air mixture containing 8% CS (the PM 2.5 particles in the air mixture was 45 ± 8.37 mg/m^3^). The mice allocated to the sham CS exposure were caged in the same environment but exposed only to fresh air for 60 min. These 60‐min CS and sham ‐CS exposure episodes were conducted twice a day, 5 days a week, for 30 weeks.

### 2.4 Interventional medication

The PDEIs dosages were determined from previous studies,^16^, ^17^, ^18^ i.e., 5 mg·kg^−1^ for ROF and 10 mg·kg^−1^ for both PTX and THEO. Determination of the TRI dosage was done by converting the equivalent dose for human beings, i.e., 24 mg TRI for a 60 kg adult, based on body surface area and adapting it accordingly for the mice.^19^ Medication was commenced from the 29th week. ROF was suspended in sterilized water and given intragastrically once a day, 5 days a week for 2 weeks while PTX and THEO were dissolved in sterilized water and given intraperitoneally once a day, 5 days a week for 2 weeks, TRI (5 mg·kg^−1^) was administered intramuscularly through a single injection at the 29th week due to its long‐lasting effect. All the mice were humanely euthanized with an overdose of sodium pentobarbitone (50 mg·kg^−1^) and exsanguinated via retinal blood vessels immediately after death at the end of 30 weeks.

### 2.5 Morphometry and biomarker assessment

The animals underwent endotracheal intubation soon after euthanasia. For further morphologic and morphometric study, one side of the lungs was fixed through intratracheal instillation of 4% paraformaldehyde (PFA) solution with a constant hydrostatic pressure of 20 cm for at least 20 min and then immersed in PFA solution for at least 4 h before further histological processing. Slices, 4 µm thick, were excised in the sagittal plane from the mid‐portion of the paraffin‐embedded lung sample and stained with hematoxylin and eosin. Morphometric studies were conducted in accordance with previously reported methods.^20^ In brief, 20 non‐overlapping fields in each slice were systemically selected using an Olympus DP72 camera system (×40 magnification power). The degree of airspace (the entire acinar air space complex, that is, alveoli and alveolar ducts combined) enlargement or emphysema was assessed by mean linear intercept (Lm, μm) with the assistance of software used for digital image evaluation (SETPanizer). The alveolar surface area (*I*_A_) was measured first; then, intercepts of alveolar septa (*P*_A_) and alveolar ducts (*P*_duct_) across a test line with a given length of D in a microscopic field were counted. Lm was calculated using the formula: Lm = *n*·D [*P*_A_ + *P*_duct_]/*I*_A_. Where *n* is the number of the microscopic fields by systemic sampling in a coherent test system. Two experts in pulmonary pathology who were blinded to the study conducted this measurement. Average of the two calculations was taken as the final measurement.

The adjacent lungs underwent intratracheal instillation of 0.5 ml of ice‐cold phosphate‐buffered saline (PBS), three times, while the contralateral main bronchus was ligated. The bronchoalveolar lavage fluid (BALF) was recovered by gentle aspiration and subjected to filtration and centrifugation at 750 g for 10 min at 4℃. The supernatant was stored at −80℃ for the subsequent measurements. The lungs of the mice not subjected to BAL or PFA fixation were excised and stored at −80℃ for HDAC‐2 assessment. The expression of IL‐8 and TNF‐α in the BALF was measured using a commercial ELISA kit (Neobioscience) according to the manufacturer's instructions.

For assessment of reactive oxygen species (ROS), the frozen sections (6 μm) of the lung tissue were stained with Dihydroethidium (DHE) (2 μmol/L) in a humidified and light‐protected chamber at 37℃ for 30 min as described.^21^ Fluorescent images were photographed under a confocal laser scanning microscope (Olympus) and the intensity of fluorescence was measured using Image‐Pro Plus 6.0^®^ (Media Cybermetics) software and expressed as arbitrary units of fluorescence. The DHE fluorescent probe combines with intracellular ROS, and leads to the production of ethidium bromide, which emits red fluorescence at the excitation light wavelength of 502 nm. The luminance of the emission is in line with the quantity of ROS produced in situ.

The nuclear components in the homogenized lung tissue were extracted using a nuclear extraction kit (Aspen). HDAC‐2 activity in the nuclear extracts was quantified using an HDAC‐2 assay kit (Epigentek Group, Brooklyn, NY) according to the manufacturer's instructions.^22^

### 2.6 Statistical analysis

Statistical analyses were performed using Graphpad Prism software 5.0 (GraphPad Software Inc.) by a statistician in a blinded manner. The test of normality was by the Kolmogorov–Smirnov method. Parametric statistics, such as ANOVA and post hoc Dunnett's comparisons, were applied if the distribution of the data was deemed normal.

### 2.7 Materials

ROF, DHE, and Dimethyl sulfoxide (DMSO) was purchased from Sigma‐Aldrich, Germany. PTX was purchased from Enzo Life Sciences, America. THEO was purchased from Medchem Express, America. Triamcinolone acetonide (TRI) was purchased from Xianju Pharmaceutical Co., Ltd.. Phosphate Buffered Saline (PBS) was purchased from HyClone.

## 3 RESULTS

### 3.1 Body weight

**FIGURE 1:**
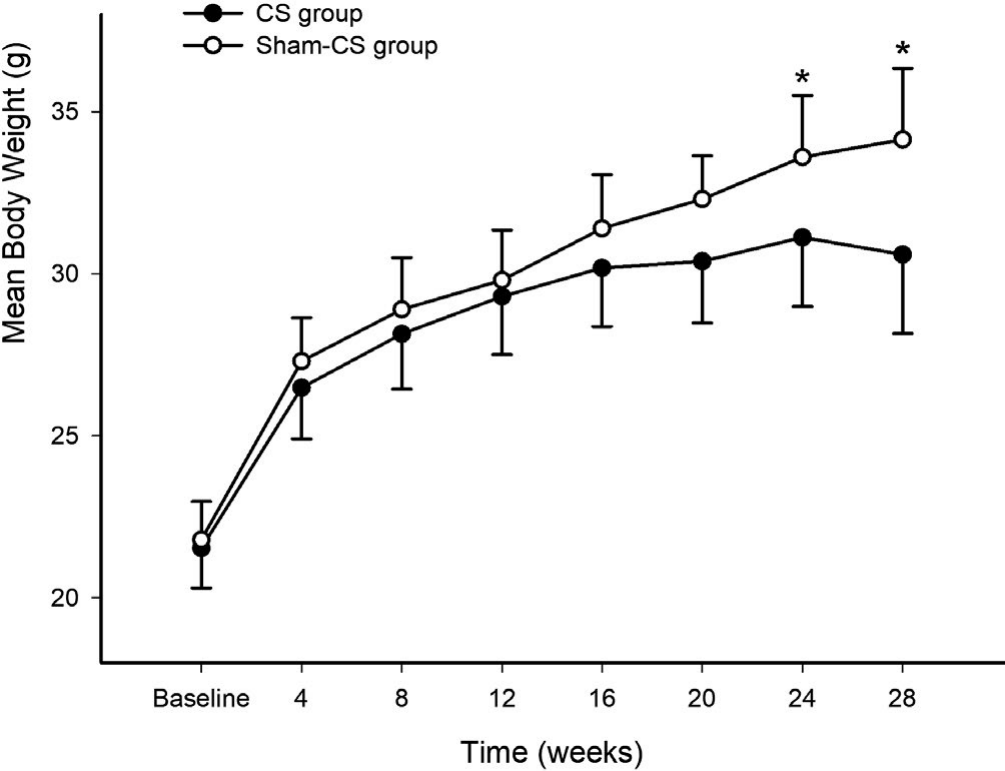
Mice with long‐term CS exposure (presented as black dots) had gained body weight slower than the animals in the sham CS exposure group (presented as circles), a significant difference in body weight was noted from the 24th week (*p* < .05). Data presented as mean body weight ± SD (*n* = 10 in each group)

The body weight gained in animals with long‐term CS exposure was slower than the animals in the sham CS exposure group. The intergroup difference in body weight attained statistical significance from the 24th week of the course (*p* < .05, Figure 1).

### 3.2 Pulmonary morphology and morphometry

**FIGURE 2:**
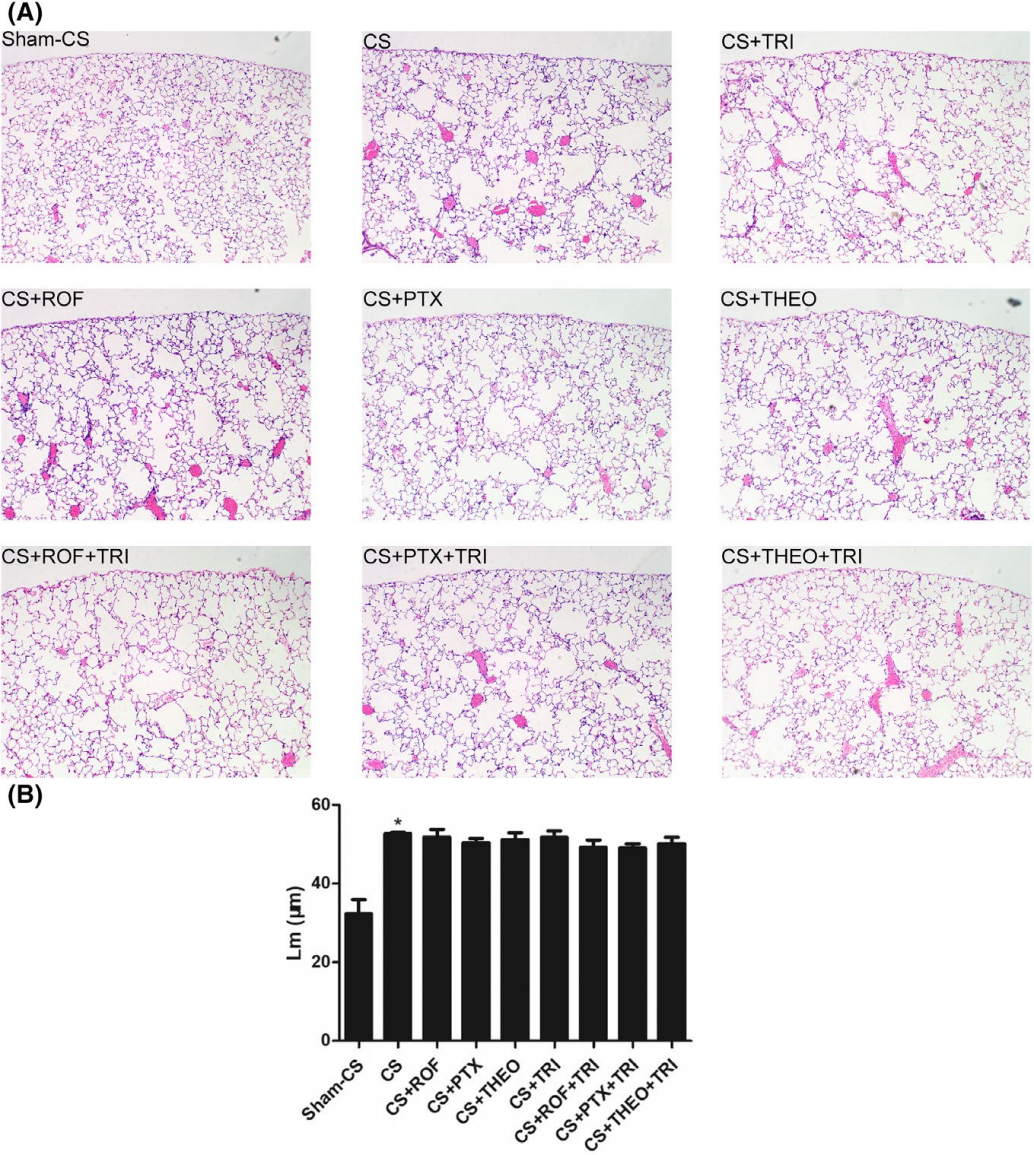
Long‐term cigarette smoke (CS) exposure induced emphysema‐like pulmonary morphometry (panel A, 10× magnification), illustrated by a greater mean liner intercept (Lm) when compared to the sham CS exposure group (panel B, *p* < .05). Two‐week course of treatment with roflumilast, pentoxifylline, theophylline, or a combination of a glucocorticoid (Triamcinolone) with any of these phosphodiesterase inhibitors, did not mitigate pulmonary emphysema (panel B, *p* > .05)

As expected, emphysema‐like structural destruction was observed in mice with long‐term CS‐exposure (Figure 2, panel A), and was associated with a greater Lm compared to the mice in sham CS exposure group (52.70 ± 0.31 μm vs. 32.39 ± 3.55 μm, *p* < .05). Two weeks’ treatment with any of these PDEIs or combined TRI with any of these PDEIs did not mitigate this structural damage (Figure 2, panel A and B, *p* > .05).

### 3.3 Pro‐inflammatory cytokines in BALF

**FIGURE 3:**
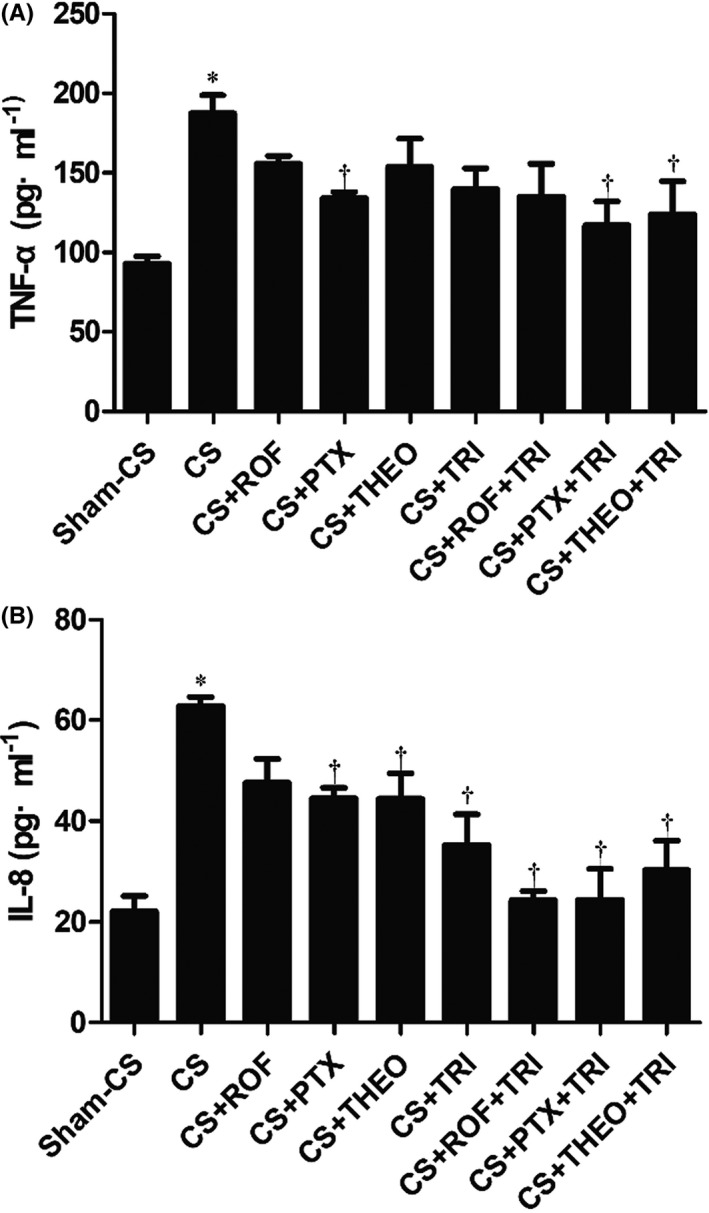
Expression of TNF‐α (panel A) and IL‐8 (panel B) in bronchoalveolar lavage fluid (BALF) samples was up‐regulated by long term cigarette smoke (CS) exposure. Only PTX down‐regulated the expression of TNF‐α, while the addition of TRI did not increase this inhibition. PTX, THEO, and TRI but not ROF down‐regulated the expression of IL‐8. The addition of TRI intensifies the inhibition, not only for PTX and THEO but also for ROF. Results presented as mean ± SD (*n* = 5 mice for each group). * *p* < .05, compared to the Sham‐CS group; ^†^ *p* < .05, compared to the CS group

Long‐term CS exposure induced the up‐regulation of TNF‐α (Figure 3, panel A) and IL‐8 (Figure 3, panel B) in BALF. The expression of IL‐8 could be down‐regulated by PTX, THEO or TRI monotherapy, whereas the expression of TNF‐α was only decreased using PTX (*p* < .05). The addition of TRI to any of these PDEIs intensified the inhibitory effect on IL‐8 (*p* < .05) but not on TNF‐α.

### 3.4 ROS expression in situ

**FIGURE 4:**
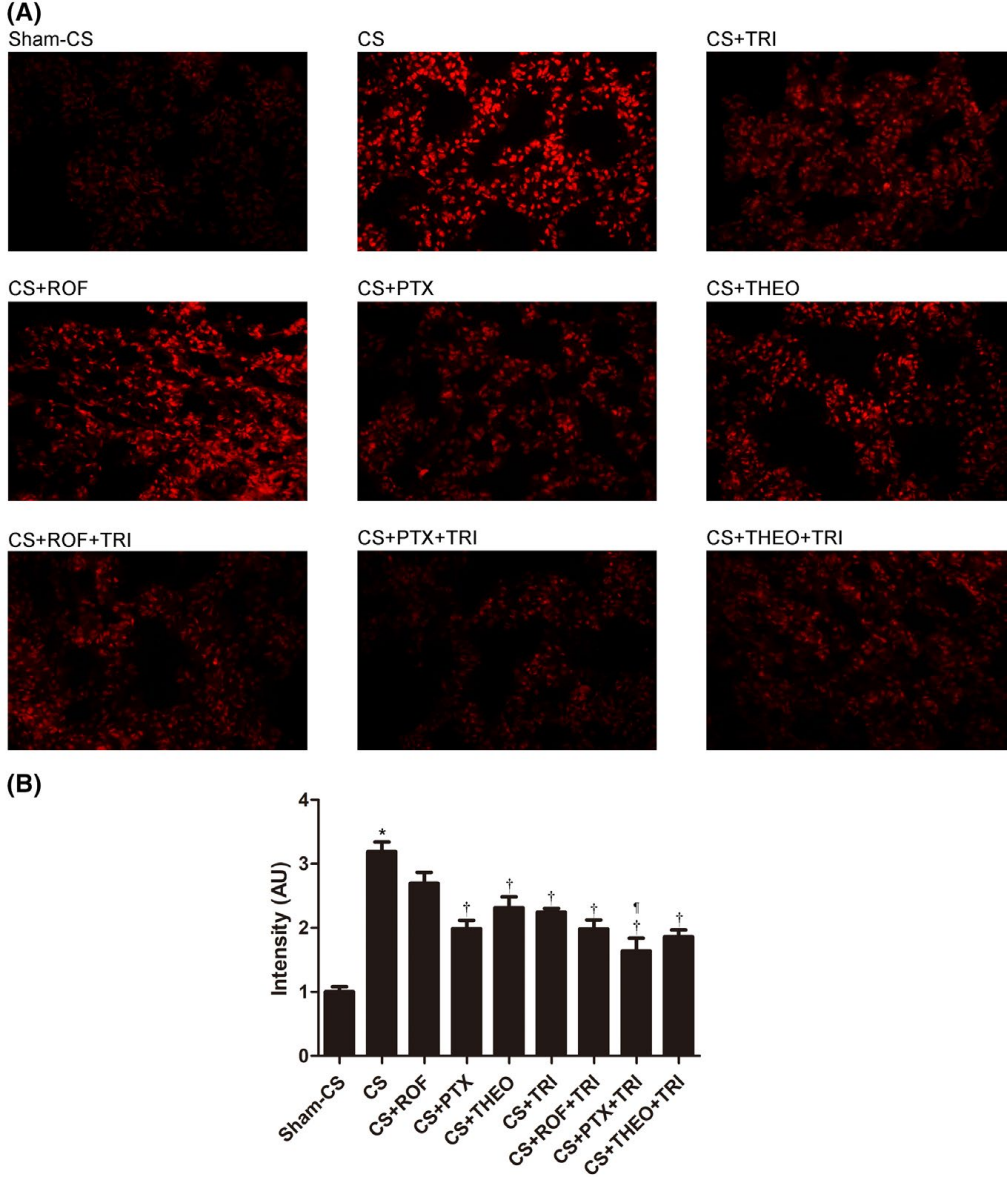
Expression of ROS in the lung tissues was augmented by long‐term CS‐exposure (panel A, *p* < .05). PTX, THEO and TRI but not ROF inhibited ROS expression. The addition of TRI to ROF, however, increased the inhibition (panel B). the addition of TRI to PTX resulted in the synergistic inhibition on ROS expression (panel B, *p* < .05). Data presented as mean ± SD (*n* = 5 mice per group). ^*^ *p* < .05, compared to the Sham‐CS group; ^†^ *p* < .05, compared to the CS group; ^¶^ *p* < .05, compared to the CS+TRI group

Expression of ROS in the lung tissue was also augmented by long‐term CS exposure (Figure 4, panel A, Table 1). The expression of ROS was decreased by the administration of PTX, THEO, or TRI but not by ROF. The addition of TRI to PTX or ROF enhanced this inhibition (Figure 4, Table 1). Combining TRI with PTX tended to be more effective than with the other PDEIs when considering ROS inhibition (Table 1, 1.64 ± 0.20 AU vs. 1.99 ± 0.14 AU, and 1.64 ± 0.20 AU vs. 1.88 ± 0.12 AU, as compared to TRI‐ROF or TRI‐THEO combination).

**TABLE 1:** Measurements of biological variables in the control and study groups (data presented as mean ± SD)

Group (*n* = 5)	Lm (μm)	TNF‐α (pg·ml^−1^)	IL‐8 (pg·ml^−1^)	ROS (AU)	HDAC‐2 (ng·μl^−1^)
Sham‐CS	32.39 ± 3.55	92.91 ± 4.65	22.01 ± 3.10	1.00 ± 0.08	29.57 ± 2.10
CS	52.70 ± 0.31*	187.39 ± 11.56*	62.79 ± 1.81*	3.19 ± 0.15*	10.99 ± 0.84*
CS + ROF	51.81 ± 1.91	155.64 ± 5.18	47.63 ± 4.76	2.70 ± 0.17	22.26 ± 2.11^†^
CS + PTX	50.44 ± 1.07	134.03 ± 3.95^†^	44.52 ± 2.11^†^	1.99 ± 0.13^†^	19.81 ± 1.18^†^
CS + THEO	51.16 ± 1.75	153.73 ± 17.87	44.46 ± 5.07^†^	2.31 ± 0.17^†^	20.82 ± 1.16^†^
CS + TRI	51.79 ± 1.63	139.59 ± 13.24	35.24 ± 6.14^†^	2.24 ± 0.06^†^	12.96 ± 1.52
CS + ROF + TRI	49.15 ± 1.94	135.00 ± 20.65	24.32 ± 1.79^†^	1.99 ± 0.14^†^	22.70 ± 1.85^†^, ^¶^
CS + PTX + TRI	49.02 ± 1.11	117.10 ± 14.92^†^	24.35 ± 6.16^†^	1.64 ± 0.20^†^, ^¶^	19.32 ± 1.25^†^, ^¶^
CS + THEO + TRI	50.07 ± 1.73	124.02 ± 20.68^†^	30.26 ± 5.88^†^	1.88 ± 0.12^†^	16.96 ± 1.33^†^

Abbreviations: CS, cigarette smoke; HDAC‐2, histone deacetylase‐2; Lm, mean linear intercept; *n* = 5 for each group; PTX, pentoxifylline; ROF, roflumilast; ROS, reactive oxygen species; THEO, theophylline; TRI, Triamcinolone acetonide.

* Compared to the counterpart variable in sham‐CS exposure control group, *p* < .01.

^†^ Compared to the counterpart variable in CS exposure control group, *p* < .05.

^¶^ Compared to the counterpart variable in CS + TRI group, *p* < .05.

### 3.5 HDAC‐2 activity in lung tissue

**FIGURE 5:**
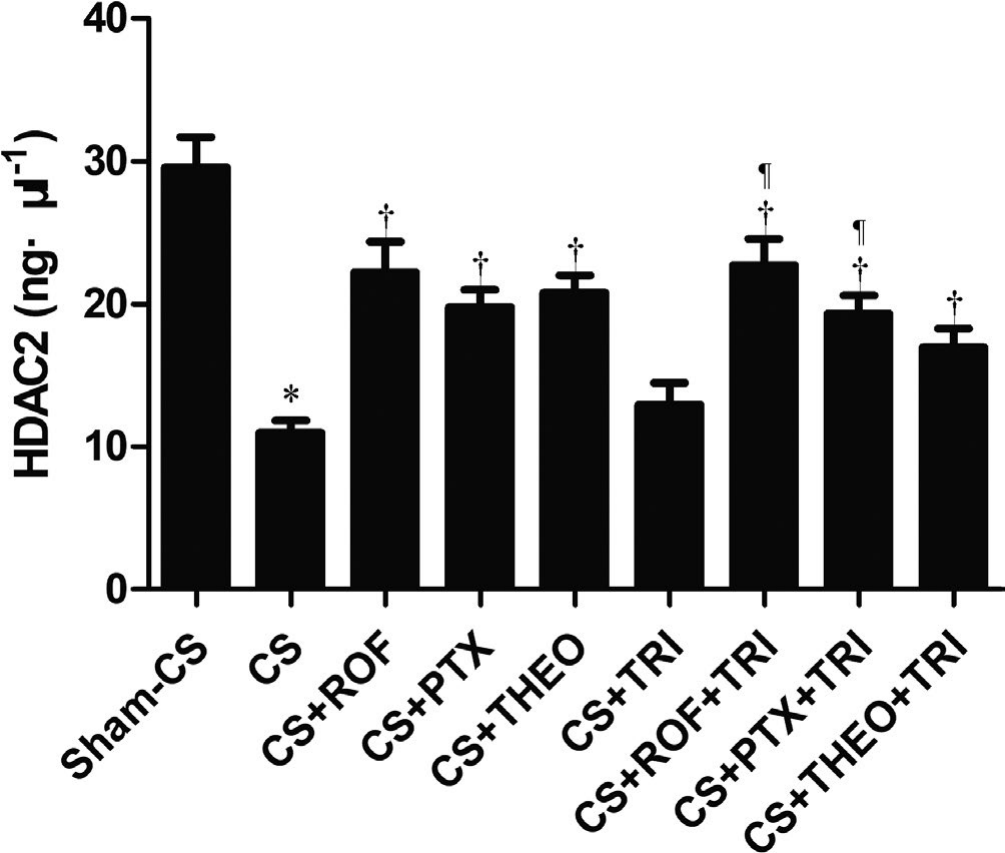
Lungs having had long‐term CS exposure had a greater reduction in HDAC‐2 activity when compared to the lungs in the sham CS exposure group. ROF, PTX, or THEO (but not TRI) partially restored HDAC‐2 activity. The addition of TRI to each of the PDEIs, however, did not enhance HDAC‐2 activity (*p* > .05). Data presented as mean ± SD (*n* = 5 mice for each group). ^*^ *p* < .05, compared to the Sham‐CS; ^†^ *p* < .05, compared to the CS group; ^¶^ *p* < .05, compared to the CS + TRI group

As shown in Figure 5 and Table 1, the lungs of mice with long‐term CS exposure had a greater reduction in HDAC‐2 activity as compared to that of the sham CS exposure group. ROF, PTX, or THEO partially restored the HDAC‐2 activity. The addition of TRI to any of these PDEIs, however, did not synergistically increase the HDAC‐2 activity further (*p* > .05).

## 4 DISCUSSION

The murine model of CS‐induced pulmonary emphysema has many similarities to human COPD, including growth delay,^23^, ^24^ aberrant inflammatory responses and augmented oxidative stress.^25^, ^26^ TNF‐α and IL‐8 were chosen as the representative inflammatory biomarkers because both play key roles in the pathogenesis of CS‐induced pulmonary inflammation.^27^, ^28^

The PDEs are classified into 11 super‐families.^11^ The involvement of PDE isoforms such as PDE3, PDE4, PDE5 and PDE7 has been demonstrated in the pathogenesis of airway inflammation and hyper‐responsiveness.^29^, ^30^, ^31^ Similar to previous studies,^17^, ^32^ the expression of ROS, IL‐8, and TNF‐α in our mice exposed to long‐term CS was elevated, which was associated with a reduction in HDAC‐2 activity and emphysema‐like pulmonary destruction. As in human COPD, in which no therapeutic modalities are able to repair pulmonary emphysematous destruction, neither the PTX nor the ROF therapy in our present study was able to mitigate the CS‐induced pulmonary damage (Lm remained unchanged) despite these PDEIs having demonstrated some anti‐inflammatory merits, that is IL‐8 expression being down‐regulated, and the HDAC‐2 activity being partially restored after 2 weeks of monotherapy with each of these PDEIs. This restoration was preferentially associated with the overall decreased expression of ROS, rather than the inhibition of any single cytokine.

PTX is the strongest anti‐inflammatory agent among the three PDEIs, if their inhibitory effects on IL‐8, TNF‐α, and ROS are considered. PTX has never been recommended for COPD treatment, as early clinical trials on PTX showed no significant clinical benefits when oxygenation and amelioration of pulmonary function in patients with COPD were taken into account.^33^, ^34^ Unfortunately, until now, no pharmacological agent has been found to reverse the natural course of COPD.^3^ Variables such as the Rate of Acute Exacerbation in a Reasonable Timespan, Speed of Lung Function Decline and Improvement of Quality of Life have been integrated as the major study endpoints in most COPD clinical studies.

Recent research unveiled that the benefit of ROF treatment in COPD is related to the inhibition of eosinophils rather than neutrophils,^35^ despite the pathogenesis of COPD being more closely related to CD8 skewness and neutrophil infiltration,^36^ suggesting that the subjects enrolled in studies concerned with the clinical efficacy of ROF, might include some patients with overlapping asthma. Clinical trials based on COPD patients using a combination of low‐dose oral THEO and inhaled corticosteroids did not show significant benefits.^15^, ^37^ PTX possesses preferential inhibitory effects on CD8 and neutrophils^38^, ^39^ and, as shown in our current study, when combined with a GC, its effect on ROS inhibition is even stronger. Therefore, the efficacy of PTX in COPD treatment should be re‐evaluated using adequate clinical variables, such as the Speed of Lung Function Decline and the Rate of Acute Exacerbation.

When considered together, we concluded that the reduction of HDAC‐2 activity is associated with the up‐regulation of ROS. THEO, as well as PTX and ROF, can restore HDAC‐2 activity by alleviating oxidative stress. Particular attention should be paid to PTX for its selective inhibitory effect on TNF‐α.

This study does have some limitations. First, CS‐induced murine pulmonary inflammation is gender and strain sensitive.^40^ Female mice are more susceptible to tobacco exposure^41^ though the male C57BL/6J strain we selected in the present study was also susceptible to CS exposure.^42^ Second, the up‐regulation of ROS only partially mirrors (as reactive nitrogen species are not evaluated) the augmented oxidative stress as a consequence of exaggerated pulmonary inflammation due to long‐term CS exposure. The mechanism that the PDEIs restore the activity of HDAC‐2 is not explained simply by referring to the dynamic changes of IL‐8, TNF‐α and oxidative stress. Third, the efficacy of the PDEIs was not evaluated with reference to pharmacokinetic and pharmacodynamic information in vivo.

## DISCLOSURE

The authors declare no conflict of interest.

## DECLARATION OF TRANSPARENCY AND SCIENTIFIC RIGOR

This declaration acknowledges that this paper adheres to the principles for transparent reporting and scientific rigor of pre‐clinical research as recommended by funding agencies, publishers and other organizations engaged with supporting research.

## Data Availability

The data that support the findings of this study are available from the corresponding author upon reasonable request. Some data may not be made available because of privacy or ethical restrictions.
